# Inhibition of O-GlcNAc transferase activity reprograms prostate cancer cell metabolism

**DOI:** 10.18632/oncotarget.7039

**Published:** 2016-01-27

**Authors:** Harri M. Itkonen, Saurabh S. Gorad, Damien Y. Duveau, Sara E.S. Martin, Anna Barkovskaya, Tone F. Bathen, Siver A. Moestue, Ian G. Mills

**Affiliations:** ^1^ Prostate Cancer Research Group, Centre for Molecular Medicine (Norway), University of Oslo and Oslo University Hospitals, Gaustadalleen, Oslo, Norway; ^2^ Department of Circulation and Medical Imaging, NTNU, Trondheim, Norway; ^3^ St. Olavs University Hospital, Trondheim, Norway; ^4^ Division of Preclinical Innovation, National Center for Advancing Translational Sciences, National Institutes of Health, Rockville, MD, USA; ^5^ Department of Microbiology and Immunobiology, Harvard Medical School, Harvard Institutes of Medicine, Boston, MA, USA; ^6^ Department of Molecular Oncology, Oslo University Hospitals, Oslo, Norway; ^7^ Department of Tumor Biology, Institute for Cancer Research, Radium hospital, Oslo University Hospital, Oslo, Norway; ^8^ PCUK/Movember Centre of Excellence for Prostate Cancer Research, Centre for Cancer Research and Cell Biology (CCRCB), Queen's University Belfast, Belfast, UK

**Keywords:** androgen receptor, prostate cancer, metabolism, glycosylation, O-GlcNAc transferase

## Abstract

Metabolic networks are highly connected and complex, but a single enzyme, O-GlcNAc transferase (OGT) can sense the availability of metabolites and also modify target proteins. We show that inhibition of OGT activity inhibits the proliferation of prostate cancer cells, leads to sustained loss of c-MYC and suppresses the expression of CDK1, elevated expression of which predicts prostate cancer recurrence (p=0.00179). Metabolic profiling revealed decreased glucose consumption and lactate production after OGT inhibition. This decreased glycolytic activity specifically sensitized prostate cancer cells, but not cells representing normal prostate epithelium, to inhibitors of oxidative phosphorylation (rotenone and metformin). Intra-cellular alanine was depleted upon OGT inhibitor treatment. OGT inhibitor increased the expression and activity of alanine aminotransferase (GPT2), an enzyme that can be targeted with a clinically approved drug, cycloserine. Simultaneous inhibition of OGT and GPT2 inhibited cell viability and growth rate, and additionally activated a cell death response. These combinatorial effects were predominantly seen in prostate cancer cells, but not in a cell-line derived from normal prostate epithelium. Combinatorial treatments were confirmed with two inhibitors against both OGT and GPT2. Taken together, here we report the reprogramming of energy metabolism upon inhibition of OGT activity, and identify synergistically lethal combinations that are prostate cancer cell specific.

## INTRODUCTION

Prostate cancer is the most common male cancer in Europe and the USA. The androgen receptor (AR), a member of the nuclear hormone receptor family, is the major target in the treatment of the disease and its function is therefore the major focus for research. Enhanced AR activity promotes cell survival and proliferation by re-programming tumour cell metabolism [1–3]. In addition, AR re-wires the metabolism of the normal prostate tissue, compared to the other differentiated cell and tissue types in the human body. The prostate gland secretes high levels of citrate, which is brought about by AR-regulated zinc accumulation to inhibit *cis*-aconitase [1]. This leads to decreased TCA cycle activity and ability to produce ATP. In the normal prostate tissue, AR additionally promotes the expression of lipogenic enzymes [4]. By doing this, AR alters the key energy producing and consuming mechanisms of the cell, and many of these changes are typically associated with proliferating cells.

In prostate cancer, the major traits of the metabolic phenotype are similar to what is seen in other cancers: increased glycolysis, abnormal phospholipid metabolism and dependence on reductive glucose metabolism [5]. It is highly likely that some of the metabolic features initially found in the normal prostate, are preserved in prostate cancer, and can be successfully targeted by repurposing metabolic inhibitors developed to treat other diseases. Indications that this is feasible have arisen from large epidemiological studies which, for example, have revealed that the use of the anti-diabetic drug metformin has strong effects on prostate cancer risk [6]. In addition, several novel compounds targeting cancer-specific metabolic abnormalities, such as glutaminase and monocarboxylate transporter 1 inhibitors, are currently assessed in clinical trials [7].

Metabolic pathways are highly inter-connected which complicates drug approaches, as normal cells can also be targeted. This challenge has led to the search for combinatorial treatment options to specifically target the vulnerabilities of cancer cells, which are not able to activate appropriate survival pathways upon dual targeting and do not cause toxicities in normal cells [8].

One of the key pathways sensing metabolic status in cells is the hexosamine biosynthetic pathway (HBP) [9]. HBP requires glucose, glutamine, acetate and UTP, and is thereby positioned to integrate information on the availability of nutrients. HBP produces the high-energy compound UDP-N-acetyl-D-glucosamine (UDP-GlcNAc), which is a sugar donor involved in synthesis of other nucleotide sugars, complex glycosylation and also utilized by O-GlcNAc transferase (OGT) to modify target proteins via single sugar conjugation [10, 11]. OGT targets key regulators of cell fate, including metabolic enzymes such as phosphofructokinase 1 [12], epigenetic regulators Oct4 and Sox2 [13] and transcriptional activity through glycosylation of the RNA Polymerase II tail to affect initiation complex formation [14]. Increased OGT expression has been detected in numerous cancers, including bladder cancer [15] and lung and colon cancers [16]. We recently reported that HBP enzymes are over-expressed in human prostate cancer patients and that three of the four HBP enzymes are induced by androgen stimulation [2, 17]. All this has triggered great interest in developing small molecule inhibitors to target OGT [18, 19].

Given the established metabolic re-programming of prostate cancer cells, we hypothesised that inhibition of OGT activity might be able to distort cancer cell metabolism. In this study, we treated prostate cancer cells with a commercially available OGT inhibitor ST045849 and analysed samples with ^1^H Nuclear Magnetic Resonance (NMR) spectroscopy. Treatment with the OGT inhibitor led to complete depletion of intracellular alanine. Gene expression analysis suggested that alanine was consumed by glutamic pyruvate transaminase 2 (alanine aminotransferase, GPT2). Combining OGT inhibition with GPT2 inhibition induced cell death specifically in prostate cancer cells.

## RESULTS

### Establishment of OGT inhibitor dose that inhibits proliferation of cancer cells

**Figure 1:**
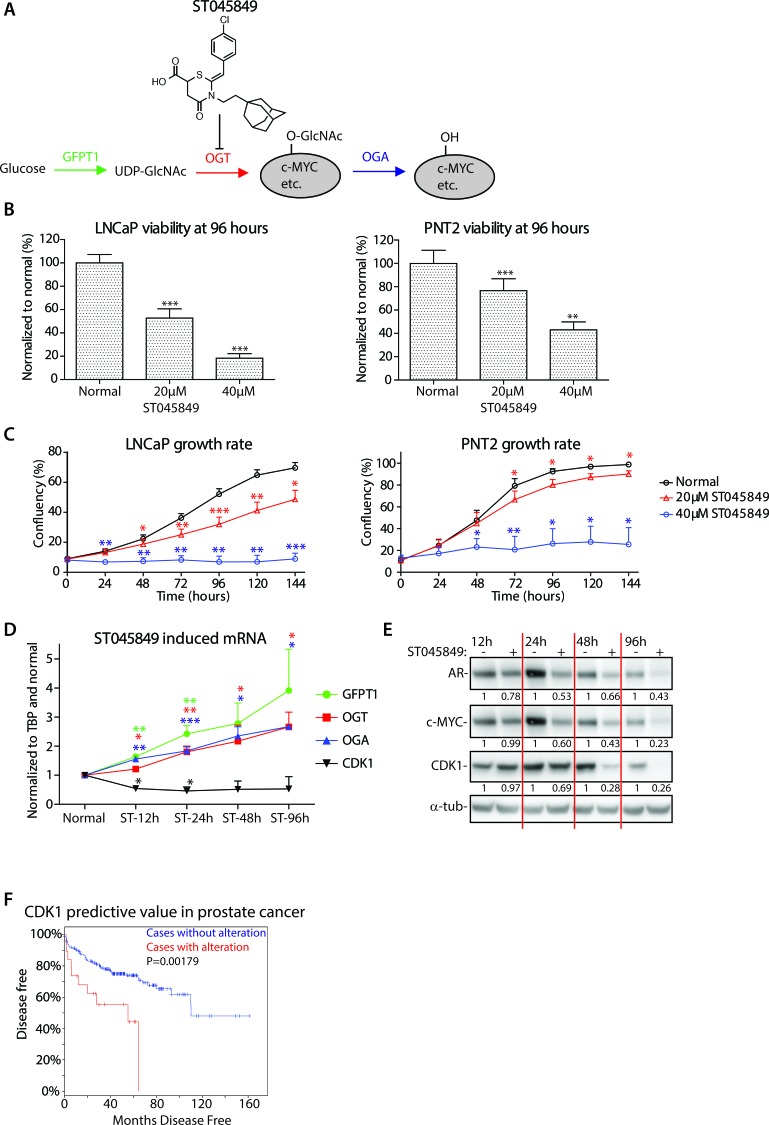
Effects of OGT inhibition on cell viability and establishment of the dose for metabolomics **A.** Enzymes regulating O-GlcNAc cycling. GFPT1 (glutamine--fructose-6-phosphate transaminase 1) is the rate-limiting enzyme in the hexosamine (HBP) biosynthetic pathway and directs glucose to the HBP rather than glycolysis. UDP-GlcNAc (UDP-N-acetylglucosamine) is the end-product of HBP and is utilized by OGT to modify target proteins *via* single sugar conjugation. c-MYC is highlighted here as one of its targets. ST045849 is a small molecule inhibitor targeting OGT. OGA (N-Acetyl-Beta-D-Glucosaminidase) removes O-GlcNAc from target proteins. **B.** LNCaP and PNT2 cells were treated with the indicated doses of OGT inhibitor ST045849 for 96 hours, and the viability was determined with the CellTiter-Glow^®^ (CTG) assay. The data shown is an average of four biological replicates and Standard Error of the Mean (SEM) is shown. The significance was assessed with Student's *t*-test, ** < 0.01 and *** < 0.001. **C.** Growth rate of cells after indicated treatments. The data shown is an average of four biological replicates with SEM. The significance was assessed with Student's *t*-test as above, * < 0.05, ** < 0.01 and *** < 0.001, red stars indicate comparison between normal and 20μM ST045849 and blue stars indicate comparison between normal and 40μM ST045849. **D.** Total mRNA was collected from LNCaP cells treated with 20μM OGT inhibitor ST045849 for 12, 24, 48 and 96 hours, and analysed with RT-qPCR. OGT inhibitor treated samples were normalized to sample without treatment at 12 hours. The data shown is an average of at least three biological replicates with SEM. The significance was assessed with Student's *t*-test (* < 0.05, ** < 0.01 and *** < 0.001) by comparing untreated sample from the corresponding time point, and each colour corresponds to the transcript measurement highlighted with the same colour. **E.** Protein lysates were harvested at 12, 24, 48 and 96 hour time-points and blotted for the markers of interest. The data shown is representative of three biological replicates. Densitometry was used to quantitate the intensity of each band, AR, c-MYC and CDK1 intensities were normalized to loading control and un-treated sample from each time-point was set to 1. **F.** Potential correlation of CDK1 expression with biochemical recurrence in prostate cancer patients was assessed using cBioPortal for Cancer Genomics (http://www.cbioportal.org/) using Taylor & al. data set [25]. Increased expression of CDK1 predicts biochemical recurrence with p value of 0.00179.

OGT activity is important for the proliferation of cancer cells, and inhibition of its activity inhibits tumour formation and metastasis [17, 20, 21]. These effects are mediated in part via down-regulation of oncogenic transcription factors such as c-MYC (Figure 1A). Previous studies have relied on transcriptomic profiling focusing on regulators of cell cycle genes. Given the prominent role of OGT as a metabolic integration point, we speculated that inhibition of its activity might additionally alter the metabolic profile of the cell.

First we wanted to identify an inhibitor dose that would modestly inhibit the proliferation of prostate cancer cells but ideally have no or low effects in cells representing normal prostate tissue. Therefore, we measured the viability of LNCaP (a prostate cancer cell line) and of PNT2 (a cell line representing normal prostate tissue) cells after 96 hours of treatment. This time-point was selected since prostate cancer cell proliferation rate is quite low and the effects on the metabolome should become clearer at late time-points. As expected, and based on previous studies [17], OGT inhibitor ST045849 significantly inhibited the viability of prostate cancer cells (Figure 1B). The same compound also decreased the viability of PNT2 cells, but to lower extent. We next evaluated the growth rate of both cell lines upon treatment with ST045849, and found that the higher dose (40μM) led to complete inhibition of proliferation in both prostate cancer cells and in cells representing normal prostate tissue (Figure 1C). Based on these data, we chose to treat cells with 20μM ST045849 to study the metabolic response of cells to OGT inhibition.

We next assessed if this dose was sufficient to decrease total O-GlcNAcylation in cancer cells, but observed that cells were able to compensate in the level of total protein O-GlcNAcylation at late time-points (data not shown), but at 1 hour after treatment with a low dose of ST045849 (20μM), we observed prominent decrease in the total protein O-GlcNAcylation (Suppl. Figure 1A). The observed compensation is not surprising, as it is well established that O-GlcNAcylation is tightly regulated [17, 22]. This compensation was mediated, at least in part, through increased expression of the rate-limiting enzyme in the hexosamine biosynthetic pathway, GFPT1 (Figure 1D and Suppl. Figure 1C, 1D). Interestingly, the expression of both OGT and O-GlcNAcase (OGA) were increased in the mRNA but not noticeably in protein level upon OGT inhibitor treatment. We next assessed whether some of the effects previously reported upon treatment with ST045849 were seen at a dose of 20μM, and observed that the compound led to sustained decrease of c-MYC at the 24, 48 and 96 hour time-points (Figure 1E) [17]. In addition, we assessed the expression levels of CDK1, the gene found to be most down-regulated by ST045849 treatment [17]. The expression of CDK1 was suppressed at 12 hours and this decreased expression was maintained until 96 hours, and the same was true in protein level (Figure 1D, 1E).

A novel OGT inhibitor, OSMI-1, was recently reported with higher specificity against OGT than other extant compounds [18, 19]. In an *in vitro* assay utilizing purified OGT, OSMI-1 has a 20-fold lower IC_50_-value once compared to ST045849. OSMI-1 has fewer side effects, and compound appears not to affect plasma-membrane glycosylation, but still requires reasonably high doses to induce effects on the total-O-GlcNAc (50μM for maximal inhibition) [19]. We first confirmed that OSMI-1 decreased total-O-GlcNAc (Suppl. Figure 1E). Treatment with OSMI-1 led maximally to 60% decrease in CDK1 mRNA (Suppl. Figure 1F). Importantly, and in agreement with ST045849-data, OSMI-1 decreased both c-MYC and CDK1 proteins by 40% at 24 and 48hours after the treatment (Suppl. Figure 1G). CDK1 phosphorylates AR and thereby stabilizes the protein and protein's transcriptional output [23]. As expected based on the reported CDK1 function, OGT inhibition also decreased AR protein expression (Figure 1E and Suppl. Figure 1G).

So far, we have established an inhibitor dose that displayed a clear decrease in the expression of an important cell cycle regulator, CDK1 [24], and a decrease in the expression of AR, a major drug target in prostate cancer. Analysis of a published prostate cancer microarray data set [25] revealed that increased expression of CDK1 predicts prostate cancer recurrence after surgery with high significance (*p* = 0.00179, Figure 1F). Based on these data, we decided to analyse the possible metabolic adaptations that enable prostate cancer cell survival despite the significant down-regulation of prominent prostate cancer oncogenes, c-MYC and AR.

### Inhibition of O-GlcNAc transferase activity inhibits glycolysis

**Figure 2:**
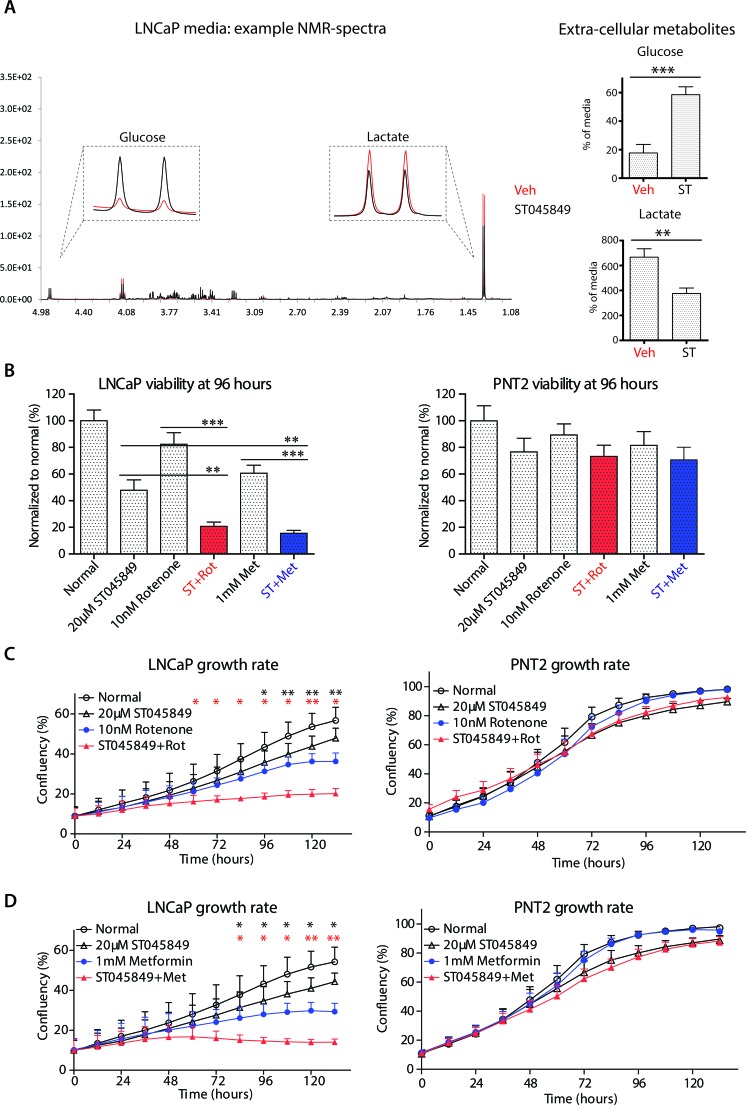
Metabolomic profiling of cell culture media after OGT inhibitor ST045849 treatment **A.** LNCaP cells were treated with 20μM OGT inhibitor ST045849 for 96 hours, cell media were collected and analyzed by ^1^H NMR. An example of the obtained NMR spectra is shown. The levels of glucose and lactate were determined from the cell culture media by ^1^H NMR. The data shown is an average of seven biological replicates with SEM. The significance was assessed with Student's *t*-test, ** < 0.01, *** < 0.001. **B.** Cells were treated as indicated in the figure and the viability of cells was analysed with CTG reagent after 96 hours treatment. Viability of the untreated sample was set to 100% and treatments were normalized to this. The data shown is an average of four biological replicates with SEM. The significance was assessed with Student's *t*-test, ** < 0.01, *** < 0.001. **C.** and **D.** Cells were treated as indicated in the figure and the growth rate of cells was recorded by life cell imaging. The data shown is an average of four biological replicates with SEM. The significance was assessed with Student's *t*-test, * < 0.05 and ** < 0.01. Black stars indicate comparison between rotenone (or metformin) only and combination of ST045849 with rotenone (or metformin), while red stars indicate comparison between ST045849 and combinatorial treatments.

Having established a dose of OGT inhibitor ST045849 for metabolic profiling, we used ^1^H NMR spectroscopy to analyse cell culture media of LNCaP prostate cancer cells treated with the OGT inhibitor. In accordance with growth inhibition, we observed a decrease in glucose consumption and in lactate production, potentially reflecting the inhibitory effects on cell growth (Figure 2A). However, we speculated that the treatment imposed a selection pressure on prostate cancer cells for a switch in metabolic dependency. Since oxidative phosphorylation can be sustained by other substrates than glucose we hypothesised that the decreased ability of these cells to cope with lower glucose uptake should make them sensitive to inhibitors of mitochondrial respiration. In order to test this hypothesis, we used two compounds: *(1)* a highly potent mitochondria complex 1 inhibitor (rotenone) at a dose of 10nM which leads to 80% decrease in complex 1 activity [26] but has only modest effect on viability, and *(2)* metformin (used at a 1mM concentration), another complex 1 inhibitor with less specificity but used in clinical setting [27]. Treatment of LNCaP cells with rotenone or metformin alone led to 20%-40% decrease in cell viability, while combining either of the compounds with the OGT inhibitor led to 80% decrease in viability (Figure 2B). We also observed near complete growth inhibition upon combinatorial treatment (Figure 2C and 2D). Interestingly, while both rotenone and metformin modestly decreased the viability and growth rates of PNT2 cells, we did not observe any additive effects with OGT inhibitor (Figure 2B-2D). These results were confirmed with the novel OGT inhibitor OSMI-1, and combinatorial treatments with either rotenone or metformin statistically significantly decreased the viability and blocked proliferation of prostate cancer cells but had no effect on cells representing normal prostate tissue (Suppl. Figure 2A-2C). In addition, treatment of another prostate cancer cell line, PC3, with either of the OGT inhibitors together with rotenone or metformin statistically significantly reduced the viability of cells (Suppl. Figure 2D).

Extracellular metabolite concentrations can reflect the energetic status of the cell, as shown here by identifying potential combinatorial treatments to inhibit proliferation. However, measuring intracellular metabolites provides a more detailed picture of the cellular metabolic networks.

### Inhibition of O-GlcNAc transferase activity leads to depletion of intracellular alanine

**Figure 3:**
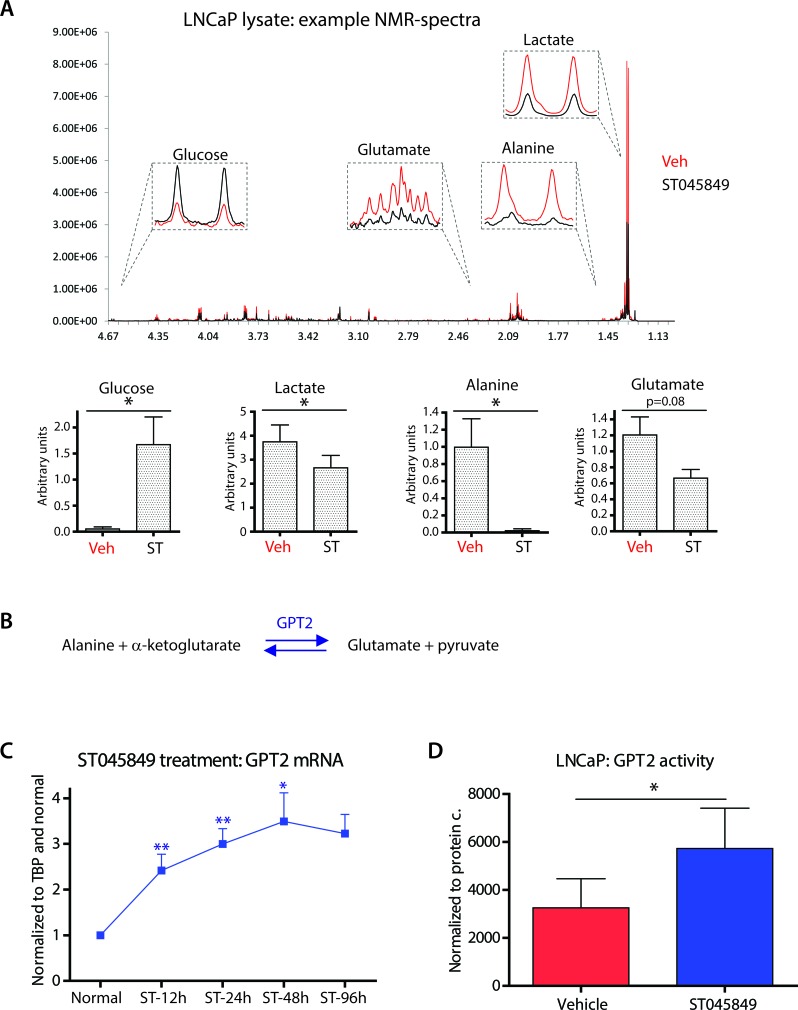
Effects of OGT inhibitor ST045849 on the levels of intracellular metabolites and enzyme activity **A.** LNCaP cells were treated with OGT inhibitor ST045849 for 96 hours, cell lysates were collected and analysed by ^1^H NMR. An example of the obtained NMR spectra is shown. Quantitation of the ^1^H NMR data. The data shown is an average of seven biological replicates with SEM. The significance was assessed with Student's *t*-test, * < 0.05. **B.** Enzymatic reaction catalysed by GPT2 (glutamic pyruvate transaminase). **C.** Total mRNA was collected from cells treated with OGT inhibitor ST045849 for 12, 24, 48 and 96 hours, and analysed with RT-qPCR. OGT inhibitor ST045849 treated samples were normalized to sample without treatment at 12 hours. The data shown is an average of at least three biological replicates with SEM and significance was assessed by comparing untreated sample from the corresponding time point, * < 0.05, ** < 0.01. **D.** Glutamic pyruvate transaminase (GPT2) assay performed from cell lysates treated either with a vehicle or with 20μM of OGT inhibitor ST045849. The GPT2 activity was assessed at 48 hours. The data shown is an average of four biological replicates with SEM. The significance was assessed with Student's *t*-test, * < 0.05.

Having established that glucose consumption is significantly decreased in prostate cancer cells treated with OGT inhibitor ST045849, we moved on to evaluate the levels of intracellular metabolites. We noted prominent accumulation of glucose and a decrease in intracellular lactate concentration, in support of decreased glycolysis (Figure 3A).

Interestingly, intra-cellular alanine was almost completely depleted in LNCaP cells and glutamate was decreased. Alanine and glutamate are inter-convertible, non-essential amino acids. At the systemic level, alanine is part of the alanine cycle, which transports carbon skeletons between muscle cells and liver, to signal for the need of increased glucose release [28]. Glutamic pyruvate transaminase (GPT2, alternative name: alanine aminotransferase) is the enzyme catalysing the metabolism of alanine (and of α-ketoglutarate) to produce pyruvate and glutamate (Figure 3B). Pyruvate can be utilized to support the TCA cycle and ATP production. In this context, alanine can be considered as an alternative energy source in the absence of sufficient glycolysis. In support of this, GPT2 is induced by starvation (Suppl. Figure 3A and 3B).

OGT inhibition increased the expression of GPT2 already at 12 hours and increased expression was maintained until 96 hours (Figure 3C). Interestingly, the intracellular alanine level was decreased by 50% after 48 hours (Suppl. Figure 3C), and the amino acid was almost completely consumed after 96 hours in LNCaP cells (Figure 3A). mRNA and metabolomics data support the hypothesis that GPT2 is activated in order to consume alanine. We next used a GPT2 activity assay to confirm that the enzyme activity is increased (Figure 3D). OGT inhibition doubled GPT2 activity, when compared to the untreated control.

Taken together, these results suggest that OGT inhibition triggers cells to rely on alanine as an alternative energy source and highlight an important role for the GPT2 enzyme.

### Inhibition of alanine aminotransferase has additive effect with OGT inhibitor

**Figure 4:**
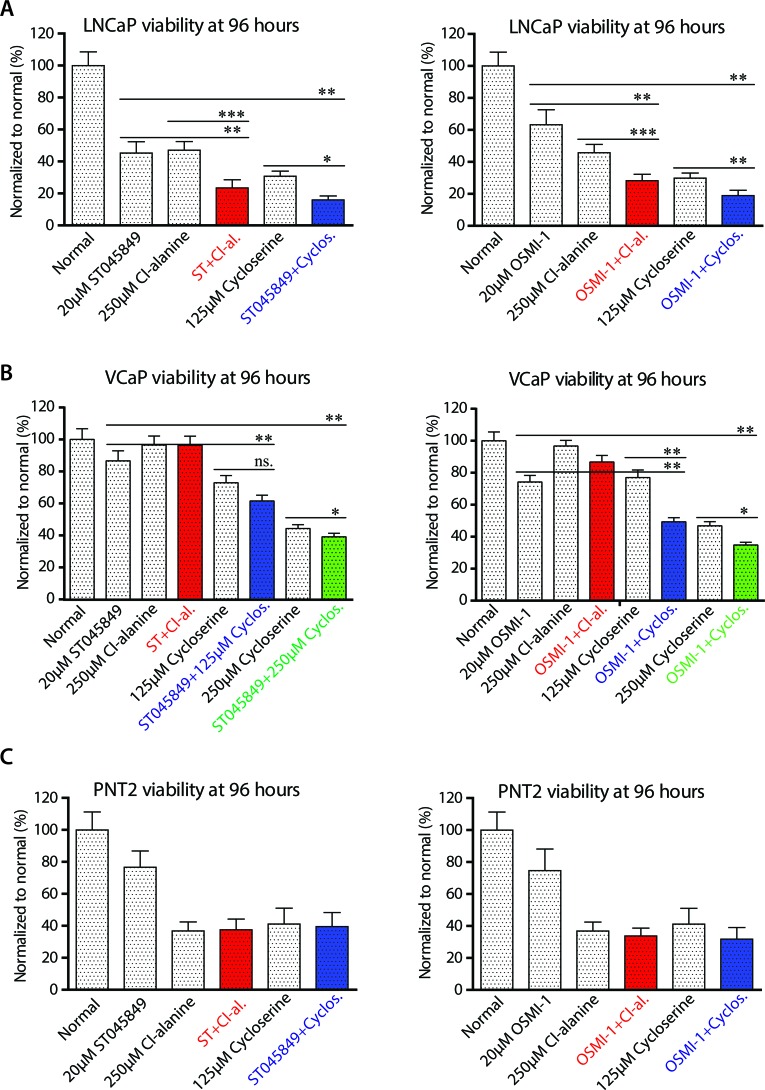
Viability of cells after combinatorial treatment with O-GlcNAc transferase and alanine aminotransferase inhibitors **A.**, **B.** and **C.** Cells were treated as indicated in the figure and the viability of cells was assessed after treatment with OGT inhibitor ST045849 or OSMI-1 alone or in combination with glutamic pyruvate transaminase (GPT2) inhibitors Cl-alanine or cycloserine. The data shown is an average of four biological replicates with SEM and significance was assessed with Student's *t*-test, * < 0.05, ** < 0.01 and *** < 0.001.

OGT inhibition led to a decrease in glucose consumption and to a significantly higher alanine consumption. In order to test whether GPT2 activity is important for cell survival upon OGT inhibition, we used two GPT2 inhibitors: chloro-alanine (Cl-alanine) and cycloserine. Combination of either of the two OGT inhibitors (ST045849 or OSMI-1) with either of the two GPT2 inhibitors led to a statistically significant decrease in the viability of LNCaP cells (Figure 4A). Both OGT inhibitors sensitized another prostate cancer cell line, VCaP, to cyclo-serine (Figure 4B). In addition, OGT inhibition sensitized a third prostate cancer cell line (PC3) to GPT2 inhibition Suppl. Figure 4A and 4B). We next confirmed the small molecule data by inhibiting OGT expression with siRNAs and treated cells with Cl-alanine (Suppl. Figure 4C). This combinatorial treatment strategy also completely blocked proliferation of LNCaP cells and inhibited proliferation of PC3 cells (Suppl. Figure 5A-5C).

We next evaluated the combinatorial treatment strategy of simultaneous targeting of OGT and GPT2 in PNT2 cells, and observed that both Cl-alanine and cycloserine alone decreased cell viability, but no additive effect was observed when both GPT2 and OGT inhibitors were combined (Figure 4C). However, combinatorial treatment decreased the growth rate of PNT2 cells, albeit to a lesser extent than LNCaP cells (Suppl. Figure 5A).

**Figure 5:**
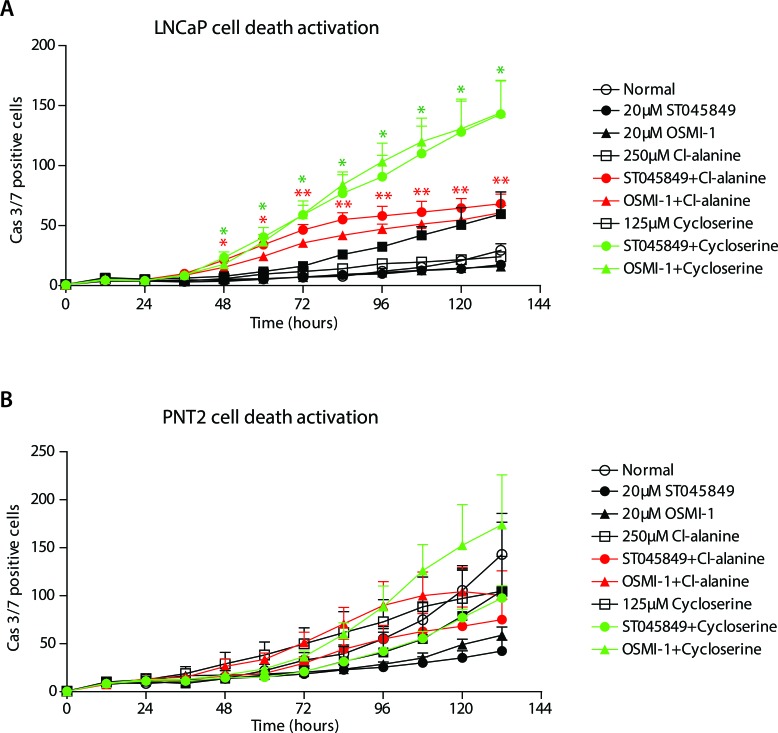
Combinatorial treatment with O-GlcNAc transferase inhibitors and alanine aminotransferase inhibitors activates cell death response in prostate cancer cells **A.** and **B.** Activation of caspases 3 and 7 was assessed in real time, imaging every 12 hours. The data shown is an average of four biological replicates with SEM and significance was assessed with Student's *t*-test, * < 0.05 and ** < 0.01. Red stars indicate comparison between either of the OGT inhibitors (ST045849 or OSMI-1) combined with Cl-alanine against any of the single treatments, while green stars indicate comparison between either of the OGT inhibitors (ST045849 or OSMI-1) combined with cycloserine against any of the single treatments.

We hypothesised that a cell line derived from normal prostate epithelium might be able to slow down proliferation due to acute deficiency in energy production, while a cell death response would be activated in prostate cancer cells. This would explain the discrepancy observed between the viability and the growth rate data. In order to test this hypothesis, we applied our combinatorial treatments to LNCaP and PNT2 cells and evaluated caspase 3/7 activation in real-time. Interestingly, caspase activation was prominently enhanced by the combinatorial treatment with OGT inhibitors and GPT2 inhibitors in LNCaP cells but not in PNT2 cells (Figure 5). Of high importance, the clinically approved drug, cycloserine, was more effective in activating the combinatorial cell death response.

## DISCUSSION

In this study, we used ST045849, a commercially available inhibitor, to study the effect of OGT inhibition on prostate cancer cell viability and metabolic reprogramming. We first established a compound dose that significantly limited the proliferation of prostate cancer cells (Figure 1B and 1C). We observed decrease in the total-O-GlcNAc levels at 1 hour after the treatment (Suppl. Figure 1A). However, total O-GlcNAc levels varied from experiment-to-experiment over time points in all conditions tested which may reflect the complex role of the HBP as a sensor of changes and stresses across a range of metabolic processes. Some of this may also at the longer time points reflect an adaptation to maintain the levels of total-O-GlcNAcylation, through increased expression of GFPT1 (Figure 1D and Suppl. Figure 1C and 1D).

Consequently, in order to understand the impact of targeting OGT on cell growth we needed to look beyond total O-GlcNAc levels and down to effects on specific genes and proteins. We speculate that proteins having only single or few sugar conjugations (such as c-MYC [29]) will show regulatory response at low doses of OGT inhibitor but this is not evident in the level of total O-GlcNAc. OGT regulates target proteins according to nutrient availability [10, 30] and growth-promoting factors need to be shut-down in the absence of nutrients to avoid cell death. However, OGT additionally has house-keeping target(s), amongst which nucleoporins (NUPs) are highly abundant [31, 32]. Complete block of OGT activity leads to decreased O-GlcNAcylation and thereby ubiquitinylation and degradation of NUPs, which compromises the nuclear pore selectivity filter [31]. Certain functions of OGT are important for the survival of normal cells, and one option to position OGT as a drug-target, is to use low dose of inhibitor and try to identify cancer-cell specific vulnerabilities.

In this work, we observed that low dose of ST045849 and OSMI-1 treatment was sufficient to decrease the expression of AR, c-MYC and CDK1 (Figure 1D and 1E and Suppl. Figure 1F and 1G). Inhibition of OGT expression has been shown to decrease the viability and invasion of prostate cancer cells through reduction of transcription factor FoxM1 [21]. Interestingly, CDK1 is required for FoxM1 transcriptional activity during cell cycle progression [33, 34]. It appears that decrease OGT activity can disrupt this positive cell cycle promoting regulatory relationship in prostate cancer cells and it remains to be determined if same is true in other cancers.

Increased CDK1 expression has not been correlated with disease progression in prostate cancer, and we show that its elevated expression increases the likelihood of prostate cancer recurrence (Figure 1F). One of the major functions of CDK1 is cell cycle regulation [24], while OGT itself directly acts on the key cell cycle regulators such as HCF-1 [35] and DNA damage response proteins [36]. OGT inhibition might sensitize cancer cells to DNA damage inducing agents, and this approach clearly demands further research.

We next performed metabolic profiling of prostate cancer cells after OGT inhibitor treatment. Treatment with ST045849 led to decreased glucose consumption and lactate production (Figure 2A). This can, at least in part, be explained through increased expression of GFPT1, to divert glucose into hexosamine biosynthetic pathway (Figure 1D). These data suggest that cancer cells decrease glycolysis due to OGT inhibition, and might be sensitized to inhibitors of oxidative phosphorylation. We confirmed this by treating cells with two different OGT inhibitors (ST045849 and OSMI-1) and with two inhibitors of oxidative phosphorylation, and observed cancer cell specific growth blockage (Figure 2B-2D and Suppl. Figure 2).

We believe that small molecule inhibitors represent the most effective therapeutic strategy to translate these findings into the clinic, even though we did observe a clear adaptation to the inhibitor treatment through increased expression of GFPT1 (Figure 1D). However, some of the desirable effects obtained through OGT inhibition, such as decrease in c-MYC, AR and CDK1, were maintained (Figure 1D and 1E).

OGT inhibition caused a near-to-complete depletion of alanine in LNCaP cells (Figure 3A). Alanine is likely consumed by GPT2 as an adaptation to OGT inhibition. Interestingly, in the absence of any inhibitor, prostate cancer cells accumulate alanine [37], and alanine level is also increased in primary samples of prostate cancer [38]. Recent advances in magnetic resonance imaging (MRI) technology may allow non-invasive, real-time assessment of alanine metabolism through hyperpolarized ^13^C labelled pyruvate, which is converted to lactate and alanine in prostate cancer[39].

GPT2 can be inhibited with small molecule inhibitors Cl-alanine and cycloserine [40]. Cycloserine is an FDA-approved drug to treat resistant forms of tuberculosis[41], but the drug also inhibits human GPT2, as shown previously [40] at similar doses as used in our study. Combinatorial treatments strongly inhibited cell viability and activated cell death response in prostate cancer cells but not in cells representing normal prostate tissue (Figures 4 and 5). Notably, GPT2 activity assays have been developed and applied successfully to blood samples [42].

In this work, we have defined complementary inhibitor targets, oxidative phosphorylation and GPT2, which can enhance response to OGT inhibition in prostate cancer cells. FDA-approved drugs such as metformin and cycloserine are available in clinical practice, thereby offering accelerated translation of these findings. OGT is also of high importance in normal cells [43], and combinatorial targeting of OGT and cancer-cell specific metabolic abnormalities may allow significant dose reduction. GPT2 functions to sustain pyruvate levels and mitochondrial activity, which enables non-invasive therapy monitoring using hyperpolarized ^13^C-pyruvate MRI. The next steps in taking OGT as a drug target into the clinic requires utilization of model organisms to better understand potential toxicities and the success of combinatorial treatment regimes.

## MATERIALS AND METHODS

### Cell lines, maintenance and treatments

Cells were obtained from ATCC and were maintained according to ATCC guidelines. For metabolomic profiling, cells were plated into either media with a commercial OGT inhibitor (ST045849, TimTec) or a vehicle control (DMSO). O-(2-Acetamido-2-deoxy-D-glucopyranosylidenamino) N-phenylcarbamate (PUGNAc), metformin, rotenone, cycloserine and Cl-alanine were obtained from Sigma. OSMI-1 compound was a gift from Professor Suzanne Walker (Harvard Medical School). Cells were allowed to attach for 1 to 3 days, and then treated as indicated in the beginning of the experiment (media was not changed at any point). OGT knockdown was performed with Lipofectamine RNAiMAX reagent according to manufacturer's instructions (Invitrogen) and OGT targeting siRNAs were obtained from Lifetechnologies (s16094 and s16095).

### Analysis of viability, growth rate and GPT2- and caspase-activity

Cells were plated into 384-well plates one day before treatment, unless otherwise stated. Viability was determined with cell titer glow (CTG) reagent (Promega). Caspase activation (CellPlayer reagent) and growth rates were determined with the IncuCyte instrument (EssenBiosciences). For GPT2 assays, two million cells were plated and activity was assessed with ALT Activity Assay (Sigma).

### Prepation of cell lysates

For NMR profiling, 350 000 cells were plated into three 6-well plate wells, and combined for analysis. Media was snap-frozen with liquid nitrogen and stored in −80°C until the analysis. Cells were collected into ethanol (−20°C). After this, cell lysates were centrifuged (18 000rpm, 10 minutes), soluble material was transferred to a new tube, evaporated with a speedvac and stored in −80°C until analysis. For mRNA profiling, mRNA was collected with illustraMiniSpin kit (GE Healthcare), reverse-transcribed with (qScript cDNA Synthesis Kit, Quanta Biosciences), and qPCR was performed with primers designed by the Primer3.0 tool (Supplementary Table 1).

### Preparation of cell lysates for western blotting

Cell lysates were prepared as described previously [17] and cell lysis buffer was supplemented with protease and phosphatase inhibitors (Roche) and PUGNAc. Membranes were probed with antibodies against AR (SantaCruz N-20 sc-816), c-MYC (Abcam ab32072), CDK1 (CST-9116), GFPT1 (CST-3818), OGT (CST- 5368), OGA (Sigma HPA036141), α-Tubulin (Millipore CP06) and GAPDH (CST-2118S).

### NMR profiling

Cell extracts were dissolved in 600 μl PBS/D2O solution containing 1.11 mM trimethylsilyl propionic acid (TSP) as a chemical shift reference. 500 μl of culture medium and blank culture medium samples (*n* = 2) were mixed with 100 μl PBS/D2O/TSP solution. The samples were transferred to 5 mm NMR tubes (Bruker Biospin GmbH, Germany) for NMR analysis at the MR Core Facility, NTNU, Trondheim, Norway, using a Bruker Avance III Ultrashielded Plus 600 MHz spectrometer (Bruker Biospin GmbH). The spectrometer was equipped with a 5 mm QCI Cryoprobe. Proton spectra were acquired using 1D NOESY (Bruker: noesygppr1d) with presaturation and spoiler gradients as described previously [44]. A standard 1 mM creatine reference solution was analyzed under identical experimental conditions and used as an external calibration standard. Pre-processing of NOESY spectra was performed using TopSpin 3.2 (Bruker BioSpin GmbH, Ettlingen, Germany). After 0.30 Hz exponential line broadening and Fourier transformation, spectra were automatically phase and baseline corrected. The chemical shift was calibrated to the TSP peak at 0.0 ppm. Peak assignments were done according to human metabolome database (HMDB; www.hmdb.ca), Chenomx NMR Suite 7.0 (Chenomx Inc., Alberta, Canada) and previously published data [45]. For cell extracts, the peak areas of lactate (1.33 ppm), alanine (1.48 ppm), glutamate (2.35 ppm) and glucose (4.64 ppm) and for culture mediums, the peak areas of lactate (1.33 ppm) and glucose (4.64 ppm) were identified and fitted as Voigt curves by polynomial regression (PeakFit v 4.12; Systat Software Inc., Chicago, IL, USA). The correlation coefficient of the fit (r^2^) for all spectra was ≥0.95. Absolute concentration of individual metabolites were calculated with reference to peak area of the standard creatine solution and the cell extract samples were normalized to the protein content while culture medium samples were normalized to the concentration of TSP, according to the PULCON principle [46].

## SUPPLEMENTARY MATERIAL TABLE AND FIGURES


