# Supplementary material for: Inhibition of O-GlcNAc transferase activity reprograms prostate cancer cell metabolism
Source: Oncotarget. 2016 Jan 27;7(11):12464–76. doi: 10.18632/oncotarget.7039 (PMC4914298; doi:10.18632/oncotarget.7039)
Supplement: Supplementary file 1 [file oncotarget-07-12464-s001.pdf]

Inhibition of O-GlcNAc transferase activity reprograms prostate cancer cell metabolism

Supplementary Material

Supplementary Table 1. Primer sequences used in the study.

**Gene name**	**Primer sequence**
OGT forward RT-qPCR	CAGCATCCCAGCTCACTT
OGT reverse RT-qPCR	CAGCTTCACAGCTATGTCTTC
OGA forward RT-qPCR	CGAGTGAACATTCCCATCACT
OGA reverse RT-qPCR	CCCAAAGGAGCACAGATGTT
GFPT1 forward RT-qPCR	CGGCTGCCTGATTTGATT
GFPT1 reverse RT-qPCR	GATAGCCTCGTCCCATTA
GPT2 forward RT-qPCR	AGCAGCCAATCACCTTCCTC
GPT2 reverse RT-qPCR	GGGCACGTTTCTTAGCATCT
CDK1 reverse RT-qPCR	GGTTCCTAGTACTGCAATTCG
CDK1 forward RT-qPCR	TTTGCCAGAAATTCGTTTGG
TBP reverse RT-qPCR	GCCAGCTTCGGAGAGTTCTG
TBP forward RT-qPCR	GCACGAAGTGCAATGGTCTTT

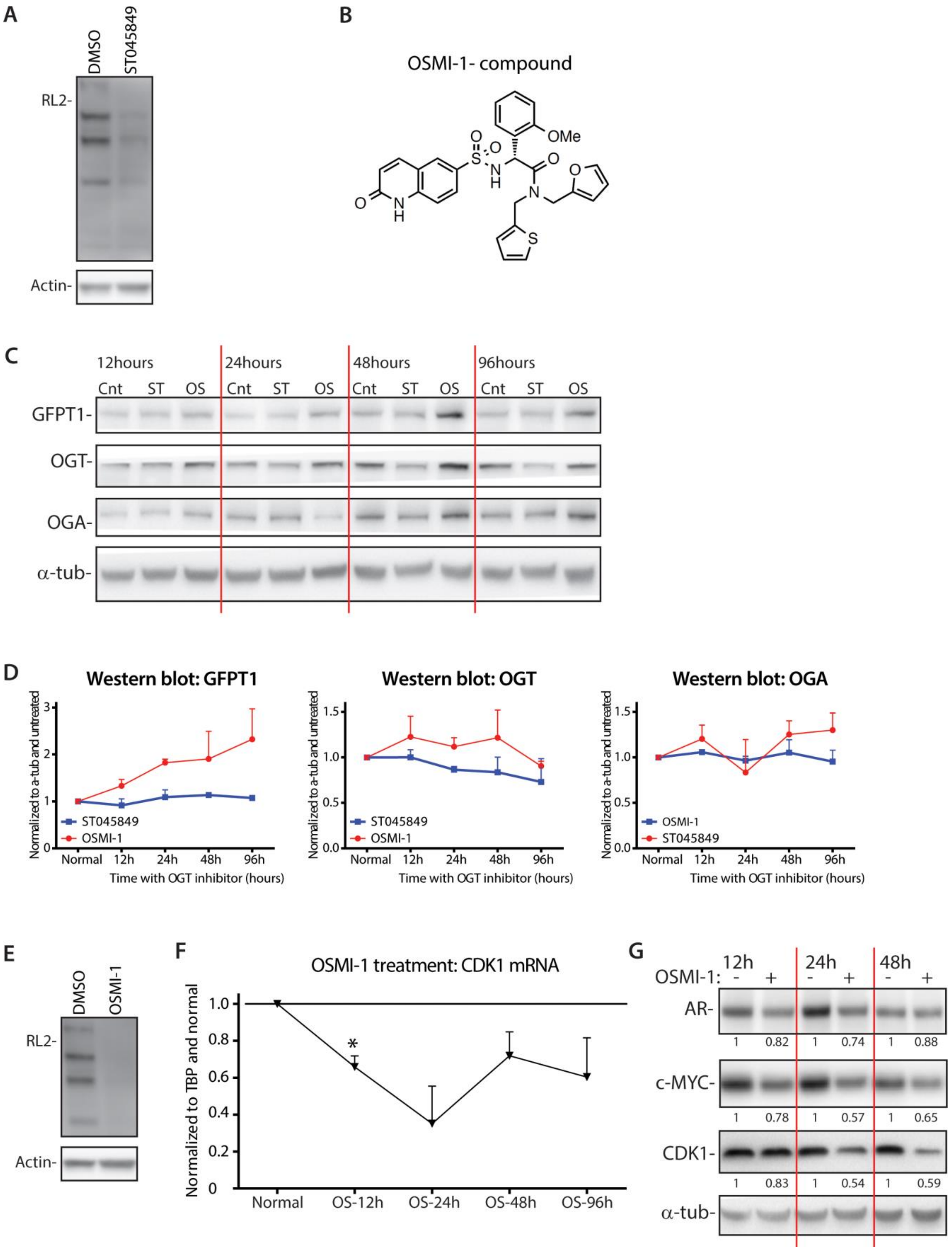

Supplementary Figure 1. A) LNCaP cells were treated either with 20µM ST045849 or vehicle control (DMSO) and harvested for western blotting at 1 hour after the treatment. **B)** Molecular

structure of the OGT inhibitor OSMI-1. **C)** LNCaP cells were treated either with 20µM ST045849 or 20µM OSMI-1 for the indicated time and harvested for Western Blotting. Data shown is representative of two biological replicates. **D)** Densitometry based quantitation of the data shown in Suppl. Fig. 1C. All the blots were normalized to loading control and untreated sample from that time-point was set to 1. **E)** LNCaP cells were treated either with 20µM OSMI-1 or vehicle control (DMSO) and harvested for western blotting at 1 hour after the treatment. **F)** Total mRNA was collected from LNCaP cells treated with 20µM OGT inhibitor OSMI-1 for 12, 24, 48 and 96 hours, and analysed with RT-qPCR. OGT inhibitor treated samples were normalized to sample without treatment at 12 hours. The data shown is an average of at least three biological replicates with SEM. The significance was assessed with Student's t-test (*<0.05). **G)** LNCaP cells were treated with 20µM OSMI-1 for the indicated time and harvested for Western Blotting. The data shown is representative of three biological replicates. Densitometry based quantitation of the data is shown below each blot. All the blots were normalized to loading control and untreated sample from that time-point was set to 1.

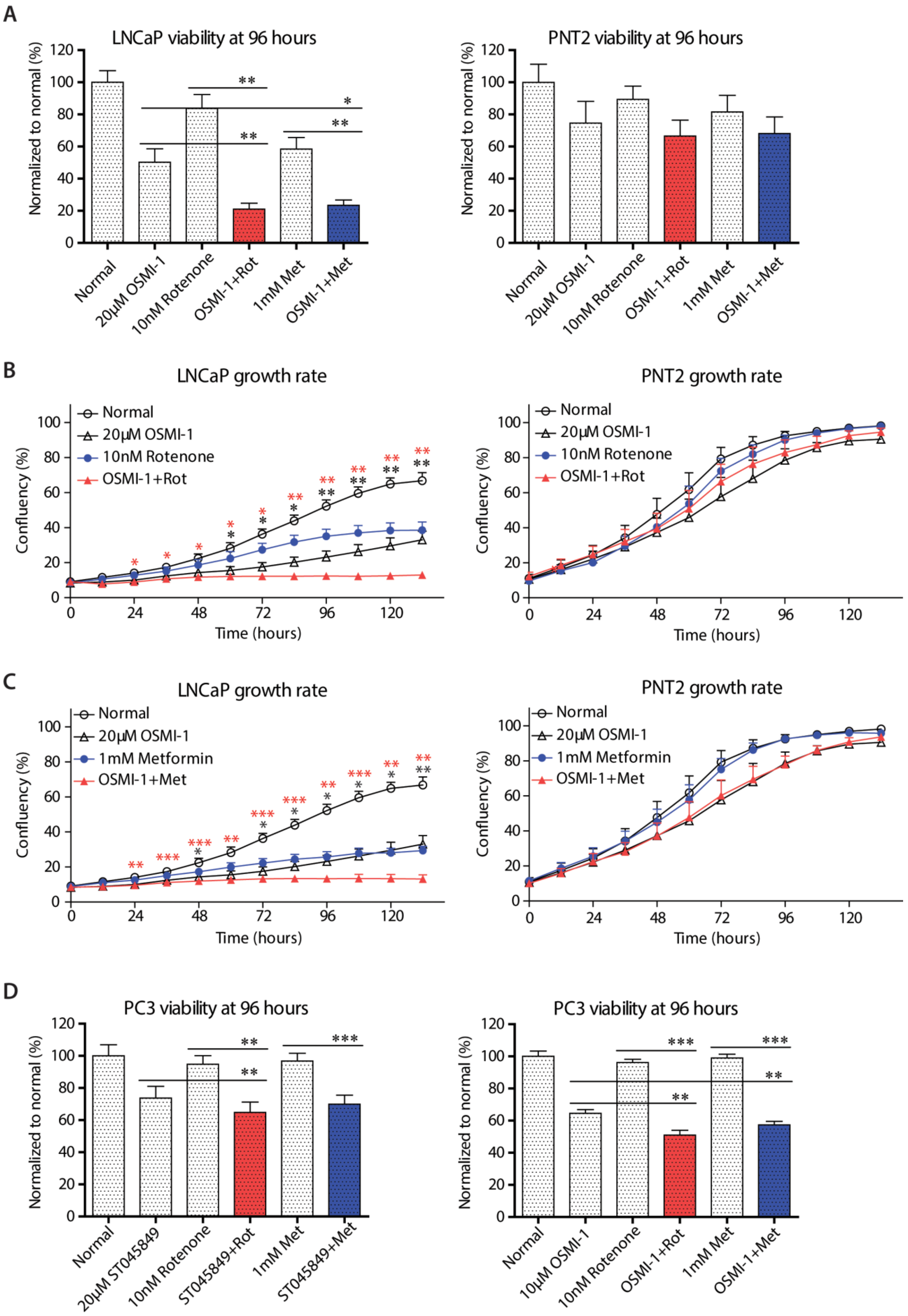
A
LNCaP viability at 96 hours
PNT2 viability at 96 hours
Normalized to normal (%)
Normal
20μM OSMI-1
10nM Rotenone
OSMI-1+Rot
1mM Met
OSMI-1+Met
B
LNCaP growth rate
PNT2 growth rate
Confluency (%)
Time (hours)
Normal
20μM OSMI-1
10nM Rotenone
OSMI-1+Rot
C
LNCaP growth rate
PNT2 growth rate
Normal
20μM OSMI-1
1mM Metformin
OSMI-1+Met
D
PC3 viability at 96 hours
PC3 viability at 96 hours
Normal
20μM ST045849
10nM Rotenone
ST045849+Rot
1mM Met
ST045849+Met
10μM OSMI-1
OSMI-1+Rot
OSMI-1+Met

Supplementary Figure 2. **A)** Cells were treated as indicated in the figure and the viability of cells was analysed with CTG reagent after 96 hours treatment. Viability of the untreated sample was set to 100% and treatments were normalized to this. The data shown is an average of four biological replicates. The significance was assessed with Student's t-test *<0.05, **<0.01. **B and C)** Cells were treated as indicated in the figure and the growth rate of cells was recorded by life cell imaging. The data is an average of four biological replicates with SEM. The significance was assessed with Student's t-test *<0.05, **<0.01 and ***<0.001. Red stars indicate comparison between rotenone (or metformin) only and combination of OSMI-1 with rotenone (or metformin), while black stars indicate comparison between OSMI-1 and combinatorial treatments. **D)** Cells were treated as indicated in the figure and the viability of cells was analysed with CTG reagent after 96 hours treatment. Viability of the untreated sample was set to 100% and treatments were normalized to this. The data shown is an average of three biological replicates. The significance was assessed with Student's t-test *<0.05, **<0.01, ***<0.001.

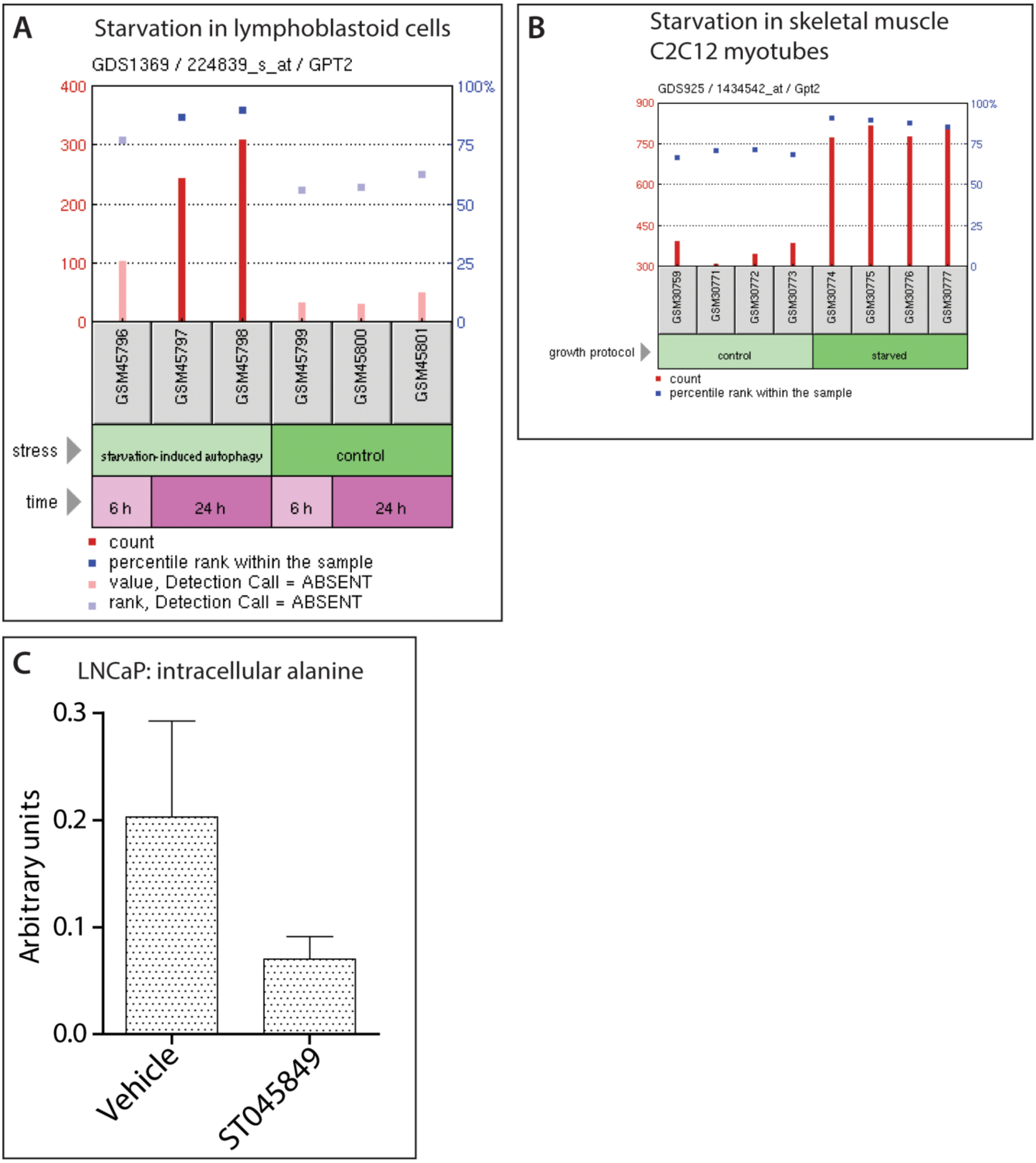

Supplementary Figure 3. The expression of alanine aminotransferase (ALAT or alternative name GPT2, glutamic pyruvate transaminase) is activated by starvation. GEO Profiles were searched for expression array experiments, in which starvation-induced gene expression profile was assessed. **A)** Human B-lymphoblastoid Awells cells were starved in Hank's Balanced Salt media for either 6 or 24 hour and total mRNA was extracted (GSE accession number GSE2435)[1]. **B)** Starvation of

mouse myocytes (GSE accession number GSE1776)[2]. Myotubes were differentiated in DMEM (supplemented with 2% horse serum) for 4 days and split in control group (media was removed and replenished every other day) and starved group (no feeding after day 4 of differentiation). After 4 days of starvation total mRNA was extracted. **C)** LNCaP cells were treated with OGT inhibitor ST045849 for 48 hours, cell lysates were collected and analysed by ^{1}H NMR spectroscopy. The data shown is an average of four biological replicates. The amount of alanine was normalized to protein content.

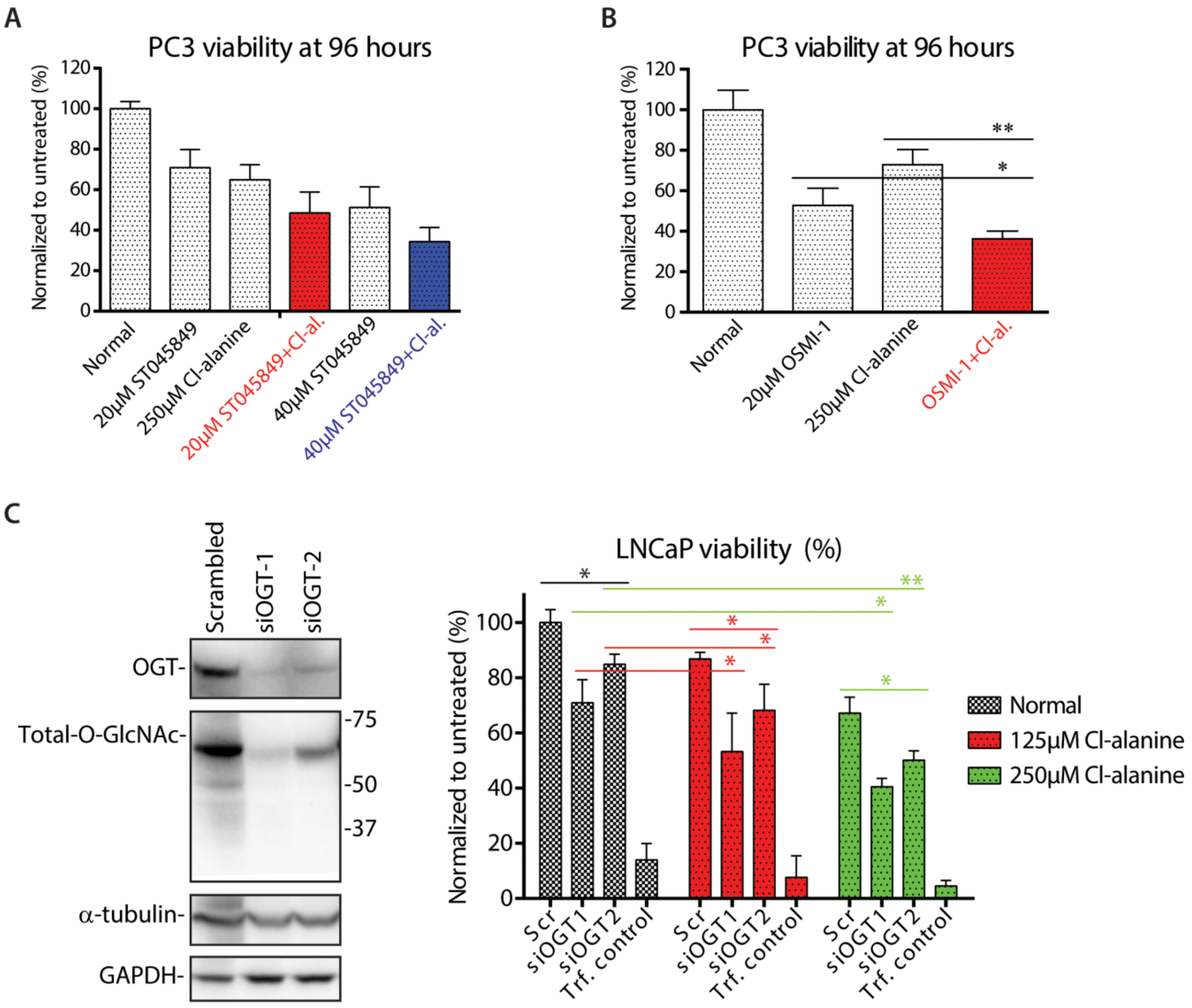

Supplementary Figure 4. **A)** Cells were treated as indicated in the figure and the viability was evaluated at 96 hours with CellTiterGlow reagent. The data shown is average of two biological replicates with SEM. **B)** Cells were treated as indicated in the figure and the viability was evaluated at 96 hours with CellTiterGlow reagent. The data shown is an average of three biological replicates with SEM. The significance was assessed with Student's t-test *<0.05 and **<0.01. **C)** The expression of OGT was inhibited with siRNA either in 6-well plates to confirm OGT knockdown by Western Blotting or in 384-well plates to measure the viability with CellTiterGlow reagent. The data is an average of three biological replicates with SEM. The significance was assessed with Student's t-test *<0.05 and **<0.01.

A

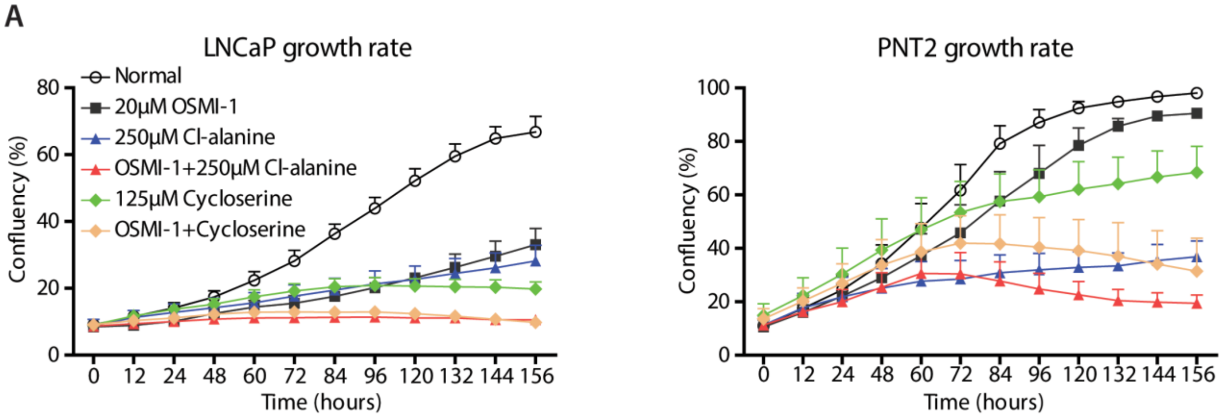

Time point	OSMI-1+Cl-al. against OSMI-1	OSMI-1+Cl-al. against Cl-al.
0	0,779266979	0,899001901
12	0,622389255	0,334924119
24	0,952289775	0,243031839
36	0,245497042	0,200714538
48	0,072929238	0,147373908
60	0,055357202	0,092021062
72	0,027802025	0,058440595
84	0,025620799	0,050336245
96	0,017218434	0,034600226
108	0,0157361	0,035518117
120	0,013570118	0,025406323
132	0,010894143	0,022272264
144	0,010386724	0,017811014
156	0,107633324	0,226218895

Time point	OSMI-1+Cyclo-S. against OSMI-1	OSMI-1+Cyclo-S. against Cyclo-S.
0	0,498680853	0,875059706
12	0,315917767	0,186268536
24	0,474489505	0,065320874
36	0,889540601	0,069034851
48	0,214297981	0,023941694
60	0,121123238	0,016691415
72	0,025960549	0,009079656
84	0,025827137	0,010875363
96	0,017914289	0,006968816
108	0,017403558	0,008286859
120	0,015088073	0,011072399
132	0,009479159	0,009627263
144	0,011040255	0,011224886
156	0,084604751	0,001890161

Time point	OSMI-1+Cl-al. against OSMI-1	OSMI-1+Cl-al. against Cl-al.
0	0,405599907	0,986158449
12	0,845939369	0,639264758
24	0,044247928	0,682197879
36	0,078822753	0,960495607
48	0,033091407	0,610835954
60	0,010703446	0,744278165
72	0,005884042	0,613548406
84	0,00390073	0,22211027
96	0,00015968	0,128652325
108	0,000121832	0,066314095
120	9,09229E-05	0,068188454
132	7,53698E-05	0,053060331
144	0,000118033	0,049549785
156	0,001475757	0,636648608

Time point	OSMI-1+Cyclo-S. against OSMI-1	OSMI-1+Cyclo-S. against Cyclo-S.
0	0,243916827	0,168634801
12	0,204540867	0,355165285
24	0,282362704	0,291594181
36	0,421861938	0,07257244
48	0,784095349	0,010901201
60	0,361665272	0,007987403
72	0,026360997	0,001459031
84	0,014640359	0,00368701
96	0,009674538	0,002543643
108	0,018675586	0,005954718
120	0,016886417	0,004127634
132	0,014730531	0,002956283
144	0,011861223	0,003409785
156	0,007576624	0,060617724

B **C**

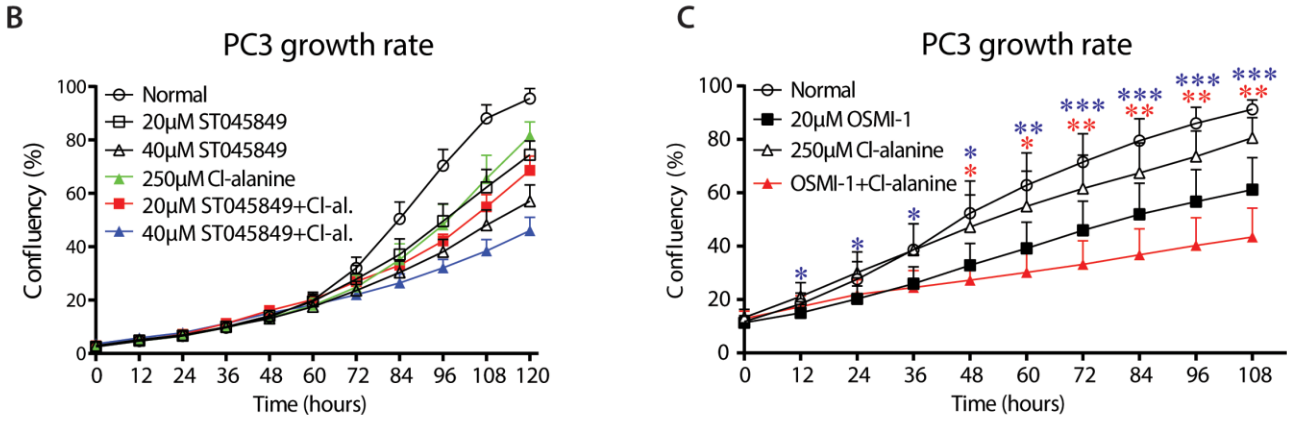

Supplementary Figure 5. **A)** Cells were treated as indicated in the figure and the growth rate of cells was recorded by live cell imaging. The data shown is an average of four biological replicates with SEM. The significance was assessed with Student's t-test and is depicted in tables below (red

indicates p-value below 0.05). **B)** Cells were treated as indicated in the figure and the growth rate of cells was recorded by live cell imaging. The data shown is representative of two biological replicates. **C)** Cells were treated as indicated in the figure and the growth rate was recorded by live cell imaging. The data shown is an average of seven biological replicates with SEM. The significance was assessed with Student's t-test *<0.05, **<0.01 ***<0.001. Blue stars refer to combinatorial treatment against Cl-alanine and red stars refer to combinatorial treatment against OSMI-1.

References

1. Dengjel J, Schoor O, Fischer R, Reich M, Kraus M, Muller M, Kreymborg K, Altenberend F, Brandenburg J, Kalbacher H, Brock R, Driessen C, Rammensee HG and Stevanovic S. Autophagy promotes MHC class II presentation of peptides from intracellular source proteins. Proceedings of the National Academy of Sciences of the United States of America. 2005; 102(22):7922-7927.

2. Stevenson EJ, Koncarevic A, Giresi PG, Jackman RW and Kandarian SC. Transcriptional profile of a myotube starvation model of atrophy. Journal of applied physiology. 2005; 98(4):1396-1406.
